# Effects of Brassinosteroid on the Physiological Changes on Two Varieties of Tea Plants Under Salt Stress

**DOI:** 10.3390/ijms252413445

**Published:** 2024-12-15

**Authors:** Zhuolu Zhang, Xiru Ma, Dandan Tang, Yiduo Chen, Guo Chen, Juanfen Zou, Liqiang Tan, Qian Tang, Wei Chen

**Affiliations:** 1 College of Horticulture, Sichuan Agricultural University, Chengdu 611130, China; zzl18380109812@163.com (Z.Z.); maxixireal@163.com (X.M.); tddtea11@163.com (D.T.); chenyiduo0201@163.com (Y.C.); 18227260416@163.com (G.C.); z18313376391@163.com (J.Z.); tanliqiang@sicau.edu.cn (L.T.); 2 Tea Refining and Innovation Key Laboratory of Sichuan Province, Chengdu 611130, China

**Keywords:** salt stress, brassinosteroids, physiology, flavonoids, theanine

## Abstract

Salt stress is one of the abiotic stresses affecting crop quality and yield, and the application of exogenous brassinosteroids (BRs) can be used in response to salt stress. However, the function of BR in tea plants under salt stress remains to be elucidated. This study investigated the effects of exogenous spraying of BR on the malondialdehyde, soluble sugar, soluble protein, and antioxidant enzyme activities in tea plants under salt stress and explored the expression changes in genes related to the synthesis pathways of proline and secondary metabolites (flavonoids and theanine). The results show that 200 mM NaCl solution inhibits the physiology of tea plants, but 0.2 mg/L BR could partially reduce the damage by increasing photosynthetic pigments, osmoregulatory substances (such as soluble sugar, soluble protein, and proline), and the activity of antioxidant enzymes (including peroxidase, catalase, and superoxide dismutase), while decreasing the malondialdehyde content in salt-stressed leaves. The qRT-PCR experiment also shows that the genes related to the synthesis pathways of proline and secondary metabolites (flavonoids and theanine) were upregulated under salt stress, and the proline degradation genes were downregulated, thus promoting the accumulation of proline under salt stress in both varieties. When tea plants were subjected to salt stress, the expression of genes related to the synthesis of secondary metabolites was regulated accordingly to resist salt stress. Moreover, spraying BR had an obvious effect on improving the salt tolerance of tea plants. Therefore, exploring a way to improve the salt tolerance of tea trees provides a reference for the subsequent study of its salt tolerance mechanism, which is of great significance for expanding the introduction area of tea trees, increasing the planting area of tea trees, and improving the yield and quality of tea.

## 1. Introduction

Soil salinization is a global ecological environment problem and one of the main factors influencing the growth and development of crops, severely restricting regional ecological safety and sustainable agricultural development. Soil salinization has a negative impact on the growth and yield of tea plants because it leads to the buildup of reactive oxygen species in plant cells, osmotic stress, and ionic toxicity. These factors inhibit physiological metabolism and photosynthesis, thereby reducing the photosynthetic efficiency and ultimately impeding the growth and development of tea plants.

Brassinosteroids (BRs) were first identified in the pollen cells of oilseed rape (*Brassica napus* L.) in 1979, and since then, they have been identified in 44 other plants, including 37 angiosperms, 5 gymnosperms, 1 alga, and 1 fern. As a natural hormone in plants, BR serves various important functions. Numerous studies have shown that BR can enhance the capacity of crops to cold [1], heat [2], drought [3,4], and salinity [5]. The results indicate that [6] the application of BR can increase the content of chlorophyll and soluble protein as well as SOD activity, and reduce MDA content. When applied to apples [7], BR led to a positive response, marked by a significant reduction in electrical conductivity and a stable Na^+^/K^+^ ratio. Additionally, the use of exogenous BR has demonstrated the potential to improve photosynthesis at low temperatures [1]. Currently, the primary focus of research on BR is on horticulture crops. BR is widely applied in enhancing the salt resistance of other vegetable crops. Moreover, the enhancement of antioxidant enzyme activity by exogenous BR has been demonstrated in chili peppers [8]. The results indicated that 0.05 mg/L BR exhibited the best increase in salt resistance, with a significant increase in the stoutness index and a marked decrease in MDA content. Zhang et al.’s [9] study on tomatoes yielded similar results, suggesting that the optimal concentration of exogenous BR varies with changes in salt stress levels. Li et al. [10] investigated the mechanism by which BR improves salt tolerance in groundnuts. Unlike previous findings, the activity of several antioxidant enzymes did not increase continuously, and there was no significant difference in SOD activity. However, the addition of exogenous BR significantly improved CAT activity compared with NaCl treatment.

As a natural hormone, BR is not only found in rapeseed plants but also in tea plants, affecting the growth and metabolism of tea plants. The use of BR has been demonstrated to alleviate salt stress in multiple ways [11], but its application and investigation in tea plants remain insufficient. So far, there have been numerous studies on the influence of BR on the photosynthesis of tea plants, especially in combination with high temperature. Wan et al. [12] suggested that BR can improve the relative photosynthetic indexes, such as Pn, Gs, Ci, and Tr. The changes in the cell structure of tea plants treated with exogenous BR indicated that the amounts of thylakoids and starch granules in chloroplasts gradually increased [13], suggesting that exogenous BR had a positive influence on the photosynthesis of tea plants and can promote the synthesis of chlorophyll, carbon assimilation, and BR biosynthesis. Meanwhile, salt stress can induce changes in the expression levels of certain genes in plants, and the expression of these genes will further promote the synthesis and accumulation of related metabolites to cope with salt stress [14]. Flavonoids are a class of aromatic compounds widely present in plants, possessing biological activities such as antioxidant, antibacterial, and anti-aging properties [15]. The synthesis of flavonoids mainly involves the phenylalanine pathway and the cinnamic acid pathway [16]. The enzymes involved, namely phenylalanine ammonia-lyase (PAL), chalcone synthase (CHS), and flavanol synthase (FLS), are highly conserved in plants, indicating that the content of flavonoids is regulated by the internal genetic mechanisms of plants [17]. The content of flavonoids is also affected by abiotic stresses like drought, salt damage, and ultraviolet radiation. Among the existing plant secondary metabolites that can help cope with salt stress, flavonoids show a relatively high response level. Induced by salt stress, the overexpression of *GSA1* occurs in rice, accompanied by an increase in the accumulation of flavonoid glycosides, consequently improving the salt tolerance of rice [18]. Under salt stress, the overexpression of *NtCHS1* in tobacco plants promoted the accumulation of rutin and enhanced the scavenging ability of reactive oxygen species, thus improving the salt tolerance of tobacco plants [19].

Investigating the changes in the physiological characteristics of tea plants under salt stress and the physiological effects of exogenously sprayed BR on salt stress will provide a reference for studying the cultivation pathways and anti-salt mechanisms to enhance the salt resistance of tea plants. Additionally, BR can alleviate the impact of abiotic stress, which has drawn increasing attention in the context of intensifying salt stress. However, the effect of brassinosteroids on salt stress in tea plants remains unclear. Therefore, this study provides a theoretical basis for evaluating the salt tolerance of tea plants, a foundation for cultivating salt-resistant tea plants, and support for using brassinosteroids to mitigate salt stress in tea plants.

## 2. Results

### 2.1. Effects of Brassinosteroids on Photosynthetic Pigments Under Salt Stress in C. sinensis

**Figure 1:**
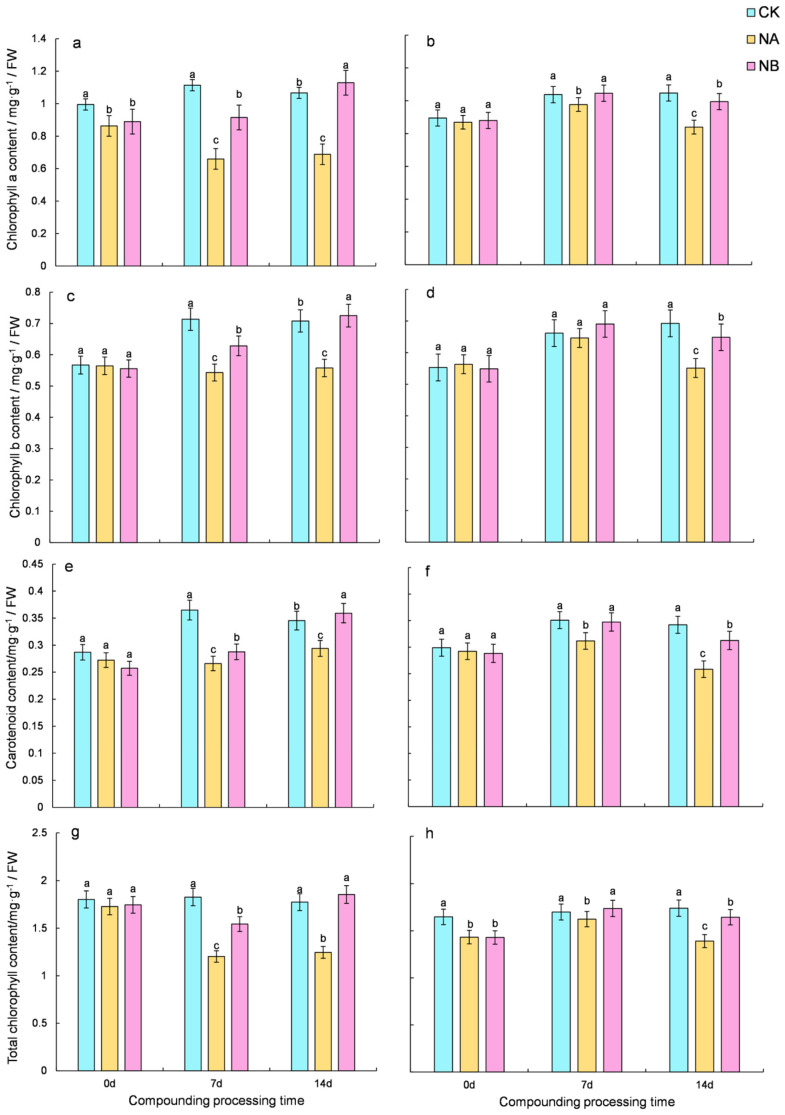
Effect of brassinosteroids on photosynthetic pigment under salt stress in tea plants. ((**a**,**c**,**e**,**g**): the content of chlorophyll a, chlorophyll b, carotenoid, and total chlorophyll in ‘FD’, respectively; (**b**,**d**,**f**,**h**): the content of chlorophyll a, chlorophyll b, carotenoid, and total chlorophyll in ‘CC’, respectively). Note: CK: normal growing tea seedlings. NA: salt-stressed tea seedlings. NB: tea seedlings subjected to salt stress and treated with BR. Different letters indicate significant differences according to Duncan’s multiple range test (*p* < 0.05).

To evaluate the impact of 0.2 mg/L BR on levels of photosynthetic pigments, specifically chlorophyll and carotenoids, under salt stress, this study monitored the changes in these pigments. Throughout the treatment period, the two tea varieties exhibited comparable trends in photosynthetic contents (Figure 1). Under salt stress (NA), the photosynthetic pigment contents decreased. In contrast, the application of 0.2 mg/L BR (NB) effectively increased the pigment levels, approaching those under normal conditions (CK).

In the salinity treatment (NA), the photosynthetic pigment contents of ‘FD’ and ‘CC’ varieties were markedly reduced. Compared with the normal conditions (CK), the total chlorophyll content of the two tea varieties decreased by 34.17% and 4.47% at 7 days and 29.8% and 20.08% at 14 days, respectively. Upon NB treatment, both ‘FD’ and ‘CC’ varieties showed a significant increase in total chlorophyll content compared to that under salt stress at 7 days and 14 days, with increases of 28.33% and 7.01% for ‘FD’ and 48.86% and 18.21% for ‘CC’, respectively (Figure 1g,h). Compared with the normal conditions (CK), the carotenoids content of the two tea varieties decreased by 27.11% and 11.13% at 7 days and 14.95% and 24.43% at 14 days, respectively. After NB treatment, both ‘FD’ and ‘CC’ varieties showed a significant increase in carotenoid content compared to that under salt stress at 7 days and 14 days, with increases of 8.14% and 11.43% for ‘FD’ and 22.26% and 20.96% for ‘CC’, respectively (Figure 1e,f).

It was observed that salt stress reduced the photosynthetic pigments in ‘FD’, and the application of 0.2 mg/L BR mitigated the loss of photosynthetic pigments in ‘FD’. The higher chlorophyll content in ‘CC’ than in ‘FD’ suggested that ‘CC’ was more salt-tolerant.

### 2.2. Effect of Brassinosteroids on MDA in Tea Plants Under Salt Stress

**Figure 2:**
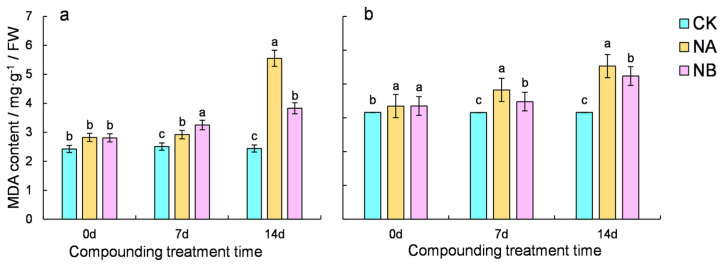
Effect of brassinosteroids on malondialdehyde (MDA) content in potted seedlings under salt stress. ((**a**): MDA content of ‘FD’; (**b**): MDA content of ‘CC’). Different letters indicate significant differences according to Duncan’s multiple range test (*p* < 0.05).

The malondialdehyde (MDA) content serves as an indicator of the degree of lipid peroxidation in plant cells. Tea plants exhibited elevated MDA levels, with ‘CC’ having a lower MDA content than ‘FD’. Notably, BR treatment reduced the MDA content in tea plants (Figure 2). Compared with the normal conditions (CK), the MDA content of the two tea varieties increased by 16.55% and 21.21% at 7 days and 127.49% and 43.50% at 14 days, respectively. Upon NB treatment, both ‘FD’ and ‘CC’ varieties showed a significant decrease in MDA content compared to that under salt stress at 7 days and 14 days, with decreases of 9.03% and 5.36% for ‘FD’ and 31.04% and 6.47% for ‘CC’, respectively. The results suggest that exogenous spraying of BR could mitigate the accumulation of malondialdehyde induced by salt stress.

### 2.3. Effect of Brassinosteroids on the Enzyme Antioxidant System of Camellia Sinensis Under Salt Stress

**Figure 3:**
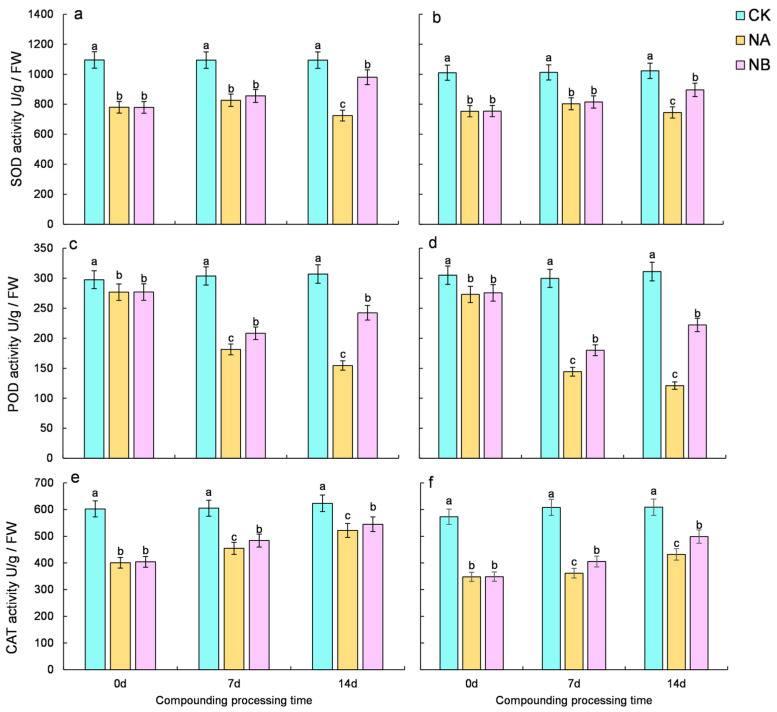
Effect of brassinosteroids on antioxidant system of potted seedlings under salt stress. ((**a**,**c**,**e**): SOD activity, POD activity, and CAT activity in ‘FD’, respectively. (**b**,**d**,**f**): SOD activity, POD activity, and CAT activity in ‘CC’, respectively). Different letters indicate significant differences according to Duncan’s multiple range test (*p* < 0.05).

As illustrated in Figure 3, salt stress induces a decline in the activity of antioxidant enzymes (POD, CAT, and SOD) in tea plants. However, following BR treatment, the overall activity of antioxidant enzymes in tea plants exhibits an upward trend. Compared with the normal conditions (CK), the SOD activity of the two tea varieties decreased by 24.46% and 20.65% at 7 days and 33.78% and 27.14% at 14 days, respectively. After NB treatment, both ‘FD’ and ‘CC’ varieties showed a significant increase in SOD activity compared to that under salt stress at 7 days and 14 days, with increases of 3.50% and 1.52% for ‘FD’ and 35.29% and 20.21% for ‘CC’, respectively. (Figure 3a,b). Compared with the normal conditions (CK), the POD activity of the two tea varieties decreased by 40.29% and 51.88% at 7 days and 49.65% and 61.09% at 14 days, respectively. Upon NB treatment, both ‘FD’ and ‘CC’ varieties showed a significant increase in POD activity compared to that under salt stress at 7 days and 14 days, with increases of 14.88% and 24.82% for ‘FD’ and 56.79% and 83.60% for ‘CC’, respectively. (Figure 3c,d). Compared with the normal conditions (CK), the CAT activity of the two tea varieties decreased by 24.84% and 40.53% at 7 days and 16.28% and 29.08% at 14 days, respectively. Following NB treatment, both ‘FD’ and ‘CC’ varieties showed a significant increase in CAT activity compared to that under salt stress at 7 days and 14 days, with increases of 6.42% and 12.10% for ‘FD’ and 4.44% and 15.50% for ‘CC’, respectively (Figure 3e,f). Overall, the activity of antioxidant enzymes in tea plants declined under salt stress, whereas it increased after BR spraying, yet failed to revert to the normal level.

### 2.4. Changes in Contents of Soluble Sugars, Soluble Proteins, and Proline

**Figure 4:**
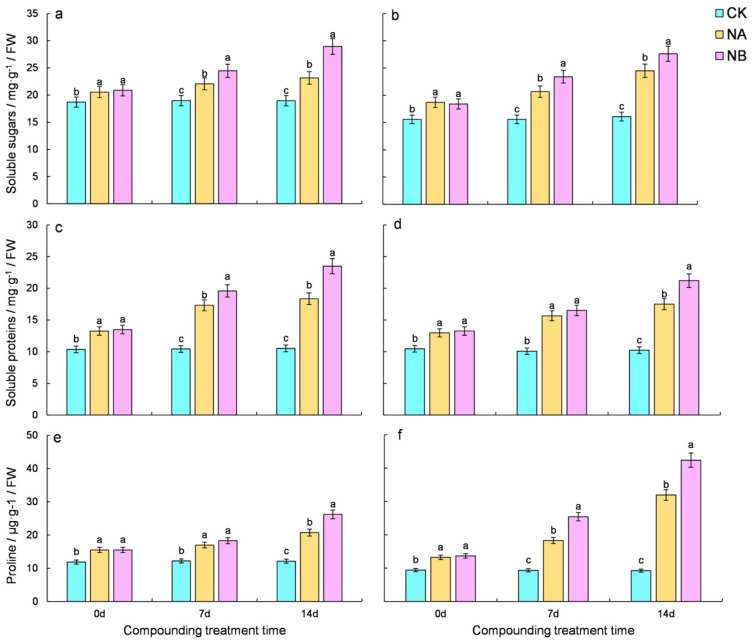
Effect of brassinosteroids on the content of Osmo modulating substances in potted seedlings under salt stress. ((**a**,**c**,**e**): soluble sugars, soluble protein, and proline in ‘FD’, respectively; (**b**,**d**,**f**): soluble sugars, soluble protein, and proline in ‘CC’, respectively). Different letters indicate significant differences according to Duncan’s multiple range test (*p* < 0.05).

Exogenous spraying of 0.2 mg/L BR under salt stress conditions led to an increase in the levels of soluble sugars, soluble proteins, and proline in both varieties. Relative to the normal control (CK), the soluble sugar content of the two tea varieties increased by 16.27% and 32.65% at 7 days and 22.14% and 52.23% at 14 days, respectively. After NB treatment, both ‘FD’ and ‘CC’ varieties showed a significant increase in soluble sugar content compared to that under salt stress at 7th day and 14th day, with increases of 10.83% and 13.20% for ‘FD’ and 25.00% and 12.76% for ‘CC’, respectively (Figure 4a,b). Compared to the normal conditions (CK), the soluble protein content of the two tea varieties increased by 66.12% and 55.48% at 7 days and 74.52% and 71.04% at 14 days, respectively. Upon NB treatment, both ‘FD’ and ‘CC’ varieties demonstrated a significant increase in soluble protein content compared to that under salt stress at 7 days and 14 days, with increases of 13.10% and 5.34% for ‘FD’ and 27.93% and 21.18% for ‘CC’, respectively (Figure 4c,d). Compared with the normal control (CK), the proline content of the two tea varieties increased by 39.17% and 95.90% at 7 days and 71.02% and 245.30% at 14 days, respectively. After NB treatment, both ‘FD’ and ‘CC’ varieties showed a significant increase in proline content compared to that under salt stress at 7 days and 14 days, with increases of 7.68% and 38.97% for ‘FD’ and 26.43% and 32.66% for ‘CC’, respectively (Figure 4e,f). However, under salt stress, the contents of soluble sugar, soluble protein, and proline in ‘CC’ were higher than those of ‘FD’ under salt stress, suggesting that NA promotes the accumulation of these substances in tea plants. Exogenous spraying of 0.2 mg/L BR has a beneficial effect on tea plants under salt stress, and this effect becomes more pronounced over time.

### 2.5. The Effect of Exogenous Brassinosteroids (BRs) on the Expression of Genes Related to the Proline Metabolism Pathway in Tea Plants Under Salt Stress

Analysis of the physiological data from this experiment revealed that proline levels were significantly affected by NA and NB treatment. By referring to the *Camellia Sinensis* ‘Shuchazao’ tea plant genome database, six genes associated with the proline metabolism pathway were identified, namely *CsProDH*-*A*, *CsProDH*-*B*, *CsP5CS*-*A*, *CsP5CS*-*B*, *CsOAT*-*A*, and *CsOAT*-*B*.

**Figure 5:**
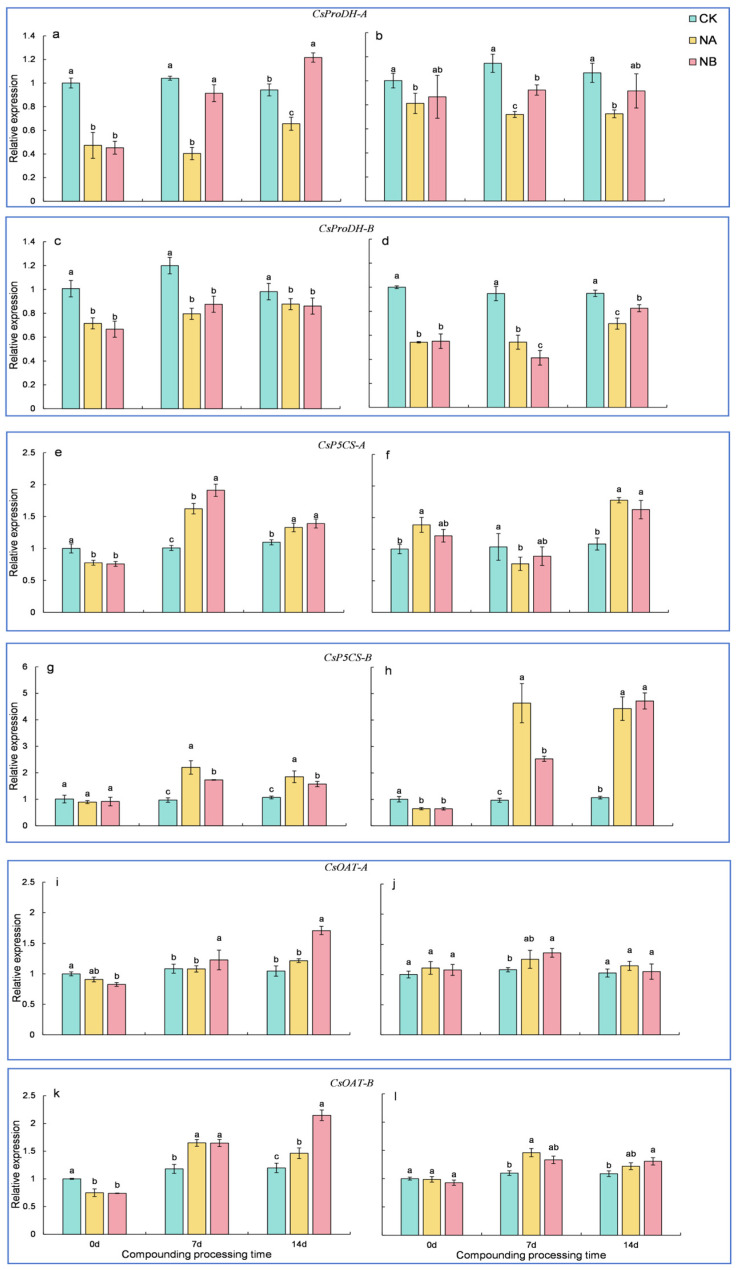
Effects of brassinosteroids on proline-related genes in potted seedlings under salt stress. ((**a**,**c**,**e**,**g**,**i**,**k**): *CsProDH*-*A*, *CsProDH*-*B*, *CsP5CS*-*A*, *CsP5CS*-*B*, *CsOAT*-*A*, and *CsOAT*-*B* in ‘FD’, respectively; (**b**,**d**,**f**,**h**,**j**,**l**): *CsProDH*-*A*, *CsProDH*-*B*, *CsP5CS*-*A*, *CsP5CS*-*B*, *CsOAT*-*A*, and *CsOAT*-*B* in ‘CC’, respectively). Different letters indicate significant differences according to Duncan’s multiple range test (*p* < 0.05).

The *CsProDH* gene encodes proline dehydrogenase (*ProDH).* Relative to the normal control (CK), the expression of *CsProDH*-*A* in the two tea varieties was downregulated by 61.20% and 37.14% at 7 days and 30.39% and 31.97% at 14 days, respectively. After NB treatment, both ‘FD’ and ‘CC’ varieties showed a significant upregulation in *CsProDH*-*A* compared to that under salt stress at 7 days and 14 days, with upregulations of 126.33% and 28.21% for ‘FD’ and 85.32% and 26.26% for ‘CC’, respectively (Figure 5a,b). Compared with the normal conditions (CK), the expression of *CsProDH*-*B* in the two tea varieties was downregulated by 33.65% and 42.69% at 7 days and 10.70% and 26.53% at 14 days, respectively. The extent of downregulation of *CsProDH*-*B* was greater in ‘CC’ than in ‘FD’. However, after NB treatment, the expression of *CsProDH*-*B* in the ‘FD’ variety showed a trend of initial upregulation followed by downregulation on the 7th and 14th days compared to that under salt stress, with rates of 10.02% and 1.84%, respectively. In contrast, the expression of *CsProDH*-*B* in the ‘CC’ variety exhibited a trend of first downregulation and then upregulation under salt stress on the 7th and 14th days, with values of 23.97% and 18.33%, respectively (Figure 5c,d). These results suggest that the expression of the *CsProDH* gene regulates the degradation of proline.

The protein encoded by the *CsP5CS* gene is pyrroline-5-carboxylate synthetase (P5CS), a key enzyme in proline synthesis in plants. Relative to the normal control (CK), the expression of *CsP5CS*-*A* in the ‘FD’ variety was upregulated by 60.80% and 21.18% at 7 days and 14 days, respectively. Under salt stress, the expression of *CsP5CS*-*A* in the ‘CC’ variety showed a trend of initial downregulation followed by upregulation on the 7th and 14 days, with rates of 25.89% and 64.05%, respectively. After NB treatment, the expression of *CsP5CS*-*A* in the ‘FD’ variety was upregulated by 17.80% and 4.64% at 7 days and 14 days, respectively. In contrast, the expression of *CsP5CS*-*A* in the ‘CC’ variety exhibited a trend of first upregulation and then downregulation under salt stress on at 7 days and 14 days, with rates of 15.77% and 8.33%, respectively (Figure 5e,f). Compared to the normal control (CK), the expression of *CsP5CS*-*B* in the two tea varieties was upregulated by 126.92% and 380.87% at 7 days and 73.16% and 316.82% at 14 days, respectively. The extent of upregulation of *CsP5CS*-*B* was greater in ‘CC’ than in ‘FD’. After NB treatment, the expression of *CsP5CS*-*B* in the ‘FD’ variety was upregulated by 21.40% and 14.88% at 7 days and 14 days, respectively. In contrast, the expression of *CsP5CS*-*B* in the ‘CC’ variety exhibited a trend of first downregulation and then upregulation under salt stress on the 7th and 14th day, with rates of 45.43% and 6.54%, respectively (Figure 5g,h).

Relative to the normal control (CK), the expression of *CsOAT*-*A* in the ‘FD’ variety was upregulated by 16.17% at 14 days. In the ‘CC’ variety, it was upregulated by 16.05% and 11.62% at 7 days and 14 days, respectively. After NB treatment, the expression of *CsOAT*-*A* in the ‘FD’ variety was upregulated by 13.57% and 40.56% at 7 days and 14 days, respectively. In contrast, the expression of *CsOAT*-*A* in the ‘CC’ variety exhibited a trend of first upregulation and then downregulation under salt stress on the 7th and 14th day, with rates of 8.53% and 8.36%, respectively (Figure 5i,j). Relative to the normal control (CK), the expression of *CsOAT*-*B* in the two tea varieties was upregulated by 39.79% and 32.90% at 7 days and 22.03% and 12.10% at 14 days, respectively. After NB treatment, the expression of *CsOAT*-*B* in the two tea varieties showed a trend of initial downregulation followed by upregulation under salt stress on the 7th and 14th day, with rates of 0.15% and 8.76% and 46.85% and 7.20%, respectively (Figure 5k,l).

Gene expression analysis revealed that the *CsP5CS* gene was initially downregulated and subsequently upregulated under salt stress, whereas the *CsProDH* gene was downregulated throughout. Regarding *CsOAT*, its expression patterns varied among different varieties under salt stress. These findings suggest that the upregulation of *CsP5CS* gene expression facilitates proline synthesis. Moreover, exogenous BR treatment promotes *CsOAT* gene expression and activates the ornithine synthesis pathway, thereby enhancing proline accumulation.

### 2.6. The Effect of Exogenous Brassinosteroids (BRs) on the Expression of Genes Related to the Flavonoid Synthesis Pathway in Tea Plants Under Salt Stress

By referring to the ‘Shuchazao’ tea plant genome database, sixteen genes associated with the flavonoid metabolism pathway were identified, namely *CsPAL*, *CsC4Ha*, *CsC4Hb*, *Cs4CL*, *CsCHSa*, *CsCHSb*, *CsF3Ha*, *CsF3Hb*, *CsF3*′*5*′*Ha*, *CsF3*′*5*′*Hb*, *CsDFRa*, *CsDFRb*, *CsLAR*, *CsANRa*, *CsANRb*, and *CsANS*. Their expression characteristics were subsequently analyzed using qRT-PCR.

**Figure 6:**
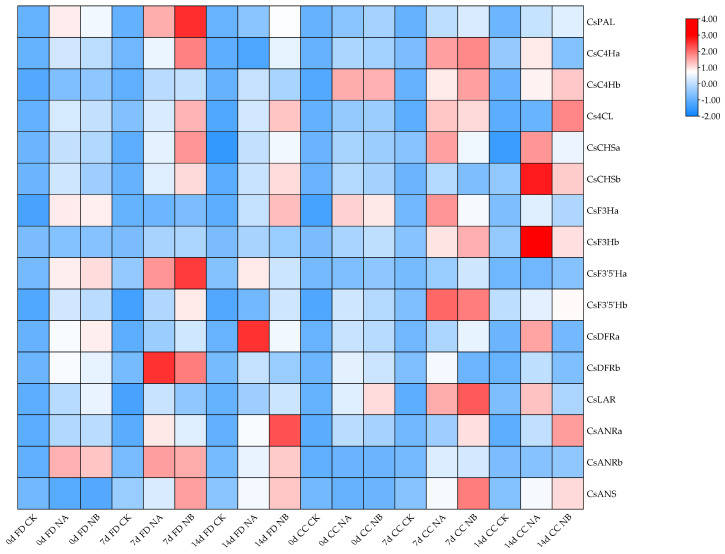
Heat map of brassinosteroids on flavonoid-synthesis-pathway-related gene expression in tea plants under salt stress. (The upregulated and downregulated genes are shown in red and blue, respectively. The scale represents the normalized peak areas of genes.).

Under salt stress treatment, all genes associated with the flavonoid synthesis pathway in both varieties exhibited an upregulation trend (Figure 6). In comparison to the salt stress treatment group, the BR spraying treatment groups also showed an upregulation trend, suggesting that BR spraying induced the expression of genes related to the flavonoid synthesis pathway, leading to an increased effect.

### 2.7. The Effect of Exogenous Brassinosteroids (BRs) on the Expression of Genes Related to the Theanine Synthesis Pathway in Tea Plants Under Salt Stress

Four genes related to the theanine synthesis pathway, namely *CsTSa*, *CsTSb*, *CsGSa*, and *CsGSb*, were identified from the ‘Shuchazao’ tea plant genome database, and their expression characteristics were analyzed using qRT-PCR.

**Figure 7:**
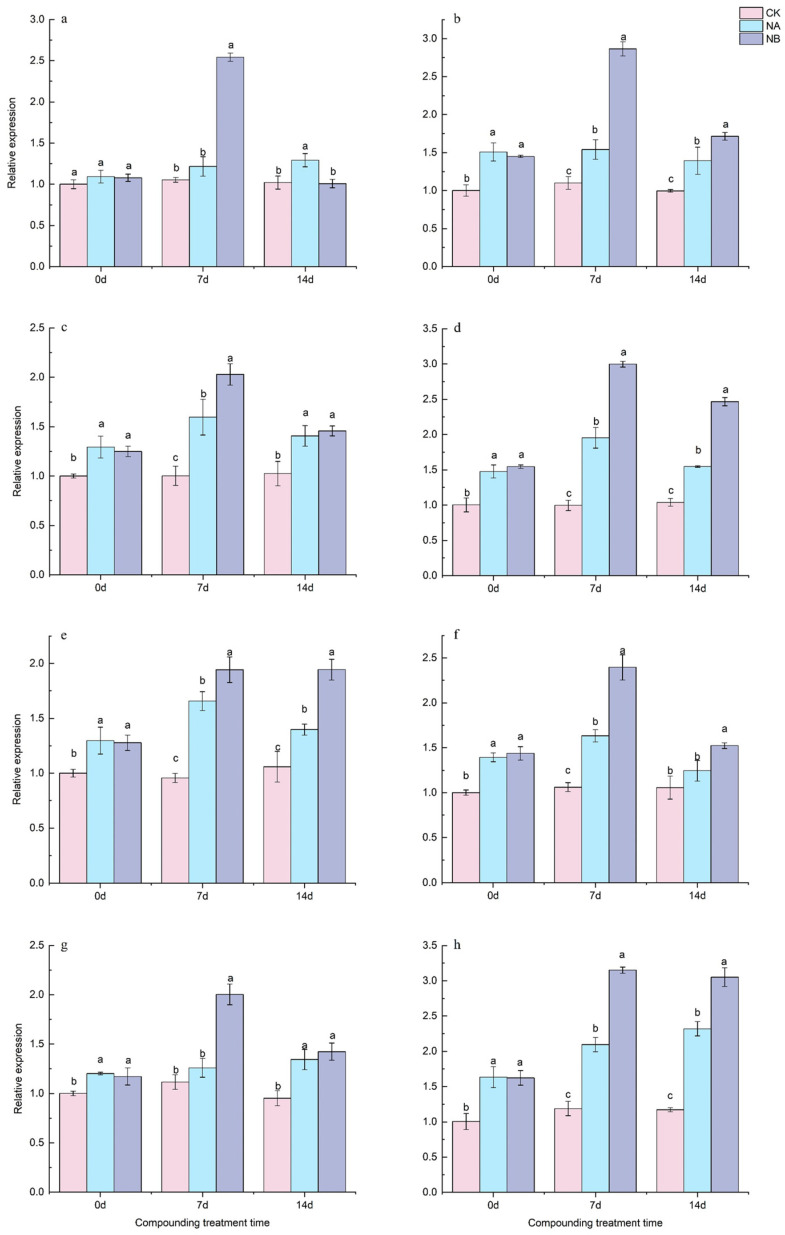
Effects of brassinosteroids on theanine-synthesis-pathway-related genes in potted seedlings under salt stress. ((**a**,**c**,**e**,**g**): *CsTSa*, *CsTSb*, *CsGSa*, and *CsGSb* in ‘FD’, respectively; (**b**,**d**,**f**,**h**): *CsTSa*, *CsTSb*, *CsGSa*, and *CsGSb* in ‘CC’, respectively). Different letters indicate significant differences according to Duncan’s multiple range test (*p* < 0.05).

As depicted in Figure 7, genes related to the theanine synthesis pathway generally exhibited an upward trend. Relative to the control (CK), both ‘FD’ and ‘CC’ varieties showed a significant increase in *CsTSa* expression at 7th day and 14th day, with upregulations of 15.52% and 26.48% for ‘FD’ and 40.07% and 39.90% for ‘CC’, respectively. After NB treatment, the expression of *CsTSa* in ‘FD’ was upregulated by 109.01% at 7th day and tended to return to the level of CK at 14th day. For ‘CC’, *CsTSa* was upregulated by 86.00% and 23.02% at 7th day and 14th day, respectively (Figure 7a,b). Compared to the control (CK), the expression of *CsTSb* in the two tea varieties was upregulated by 59.37% and 37.23% at 7 days and 96.13% and 49.09% at 14 days, respectively. After NB treatment, both ‘FD’ and ‘CC’ varieties showed a significant increase in *CsTSb* expression at 7th day and 14th day, with upregulations of 27.07% and 3.58% for ‘FD’ and 53.30% and 59.36% for ‘CC’, respectively (Figure 7c,d). 

Relative to the control (CK), both ‘FD’ and ‘CC’ varieties showed a significant increase in *CsGSa* expression at 7th day and 14th day, with upregulations of 73.26% and 32.02% for ‘FD’ and 53.87% and 17.83% for ‘CC’, respectively. After NB treatment, both ‘FD’ and ‘CC’ varieties exhibited a significant increase in *CsGSa* expression at 7th day and 14th day, with upregulations of 17.17% and 39.01% for ‘FD’ and 46.70% and 22.45% for ‘CC’, respectively (Figure 7e,f). Compared to the control (CK), the expression of *CsGSb* in the two tea varieties was upregulated by 12.94% and 41.21% at 7th day and 76.27% and 98.05% at 14th day, respectively. After NB treatment, the expression of *CsGSb* in the two tea varieties was upregulated by 59.02% and 5.94% at 7th day and 50.34% and 31.52% at 14th day, respectively (Figure 7g,h).

The *CsTS* gene is closely associated with theanine synthesis in tea plants. When tea plants are exposed to salt stress, *CsTS* is induced to express, potentially leading to increased theanine synthesis. Overall, both *CsTS* and *CsGS* genes were upregulated under salt stress. After BR spraying, an enhancing effect was observed, and their expression levels gradually returned to normal at 14th day.

### 2.8. Genes Related to Stress

Four adversity marker genes, namely *CsRD29A*, *CsRD29B*, *CsRD20*, and *CsADH1*, were identified from the genome database of *Camellia sinensis* ‘Shuchazao’, and their expression characteristics were analyzed using qRT-PCR.

**Figure 8:**
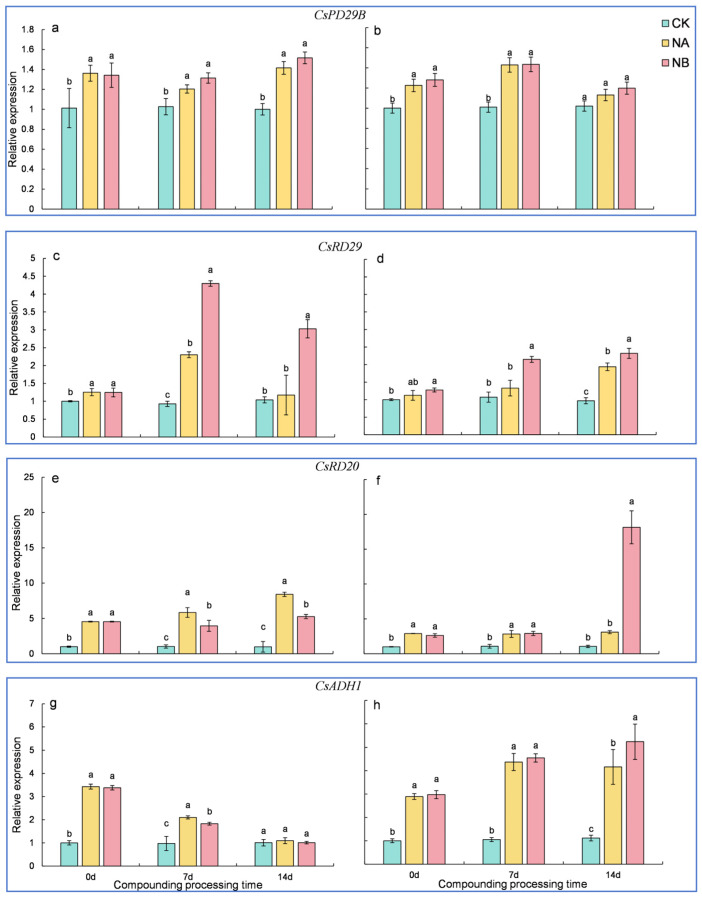
Effects of brassinosteroids on stress genes in potted seedlings under salt stress. ((**a**,**c**,**e**,**g**): *CsRD29B*, *CsRD29A*, *CsRD20*, and *CsADH1* in ‘FD’, respectively; (**b**,**d**,**f**,**h**): *CsRD29B*, *CsRD29A*, *CsRD20*, and *CsADH1* in ‘CC’, respectively). Different letters indicate significant differences according to Duncan’s multiple range test (*p* < 0.05).

As shown in Figure 8, the expression of genes associated with stress significantly increased upon exposure to salt stress (NA) and compound treatment (NB). Relative to the normal control (CK), both ‘FD’ and ‘CC’ varieties showed a significant upregulation in *CsRD29B* expression at 7th day and 14th day, with upregulations of 17.25% and 41.38% for ‘FD’ and 41.42% and 10.72% for ‘CC’, respectively. After NB treatment, the expression of *CsRD29B* in the two tea varieties was upregulated by 9.17% and 0.39% at 7th day and 7.17% and 6.00% at 14th day, respectively (Figure 8a,b). Compared to the control (CK), the expression of *CsRD29A* in the two tea varieties was upregulated by 148.27% and 24.05% at 7th day and 12.89% and 100.01% at 14th day, respectively. After NB treatment, both ‘FD’ and ‘CC’ varieties showed a significant upregulation in *CsRD29A* expression at 7th day and 14th day, with upregulations of 86.59% and 61.60% for ‘FD’ and 158.10% and 19.75% for ‘CC’, respectively (Figure 8c,d). The extent of *CsRD29A* upregulation was higher in ‘FD’ than in ‘CC’. Generally, under the composite treatment, *CsRD29A* levels in both varieties were significantly higher than those in other treatment groups, implying that this gene plays a crucial role in the response to salt stress when sprayed with 0.2 mg/L BR. Additionally, the expression level of *CsRD29A* in the combined treatment was higher than that under salt stress alone, suggesting that BR can enhance the plant’s response to salt stress. The *CsRD29B* gene in ‘CC’ also responds to salt stress, albeit less prominently than *CsRD29A*.

Relative to the control (CK), both ‘FD’ and ‘CC’ varieties showed a significant upregulation in *CsRD20* expression at 7th day and 14th day, with upregulations of 463.94% and 165.92% for ‘FD’ and 748.97% and 193.37% for ‘CC’, respectively. The extent of *CsRD20* upregulation was higher in ‘FD’ than in ‘CC’. After NB treatment, the expression of *CsRD20* in ‘FD’ was downregulated by 32.39% and 37.48% at 7th day and 14th day, respectively. In contrast, the expression of *CsRD20* in ‘CC’ was upregulated by 2.69% and 486.53% at 7th day and 14th day, respectively (Figure 8e,f). Overall, *CsRD20* acts as a rapid early responder. In ‘FD’ plants, exogenous BR treatment alleviates its high expression, whereas in ‘CC’ plants, the gene response is slower. Additionally, the *CsADH1* gene exhibits differential expression patterns across varieties. 

Relative to the control (CK), both ‘FD’ and ‘CC’ varieties exhibited a significant upregulation in *CsADH1* expression at 7th day and 14th day, with upregulations of 114.82% and 315.43% for ‘FD’ and 8.74% and 272.41% for ‘CC’, respectively. The extent of *CsADH1* upregulation was higher in ‘CC’ than in ‘FD’. After NB treatment, the expression of *CsADH1* in ‘FD’ was downregulated by 12.95% and 7.76% at 7th day and 14th day, respectively, while that in ‘CC’ was upregulated by 4.02% and 25.71%. At the same time points, ‘FD’ and ‘CC’ varieties exhibited a more significant enhancement than CK at 7 d and 14 d in *CsADH1*, and expression was upregulated by 114.82% and 315.43% and 8.74% and 272.41%, respectively. The upregulation of *CsADH1* in ‘CC’ is higher than that in ‘FD’. Under NB treatment, *CsADH1* expression in the ‘FD’ variety at 7 d and 14 d was downregulated by 12.95% and 7.76%. *CsADH1* expression in the ‘CC’ variety at 7 d and 14 d was upregulated by 4.02% and 25.71% (Figure 8g,h).

## 3. Discussion

This study focused on exploring the impact of brassinosteroids (BRs) on plants’ response to salt stress. Chlorophyll biosynthesis represents a crucial biological process in plants, and chlorophyll content is among the most significant factors influencing tea yield and quality [20]. For instance, in rice seedlings, treatment with 0.2 mg/L BR led to an increase in chlorophyll levels, suggesting an enhancement in the photosynthetic efficiency of the seedlings. Analogous results have been reported in tomatoes, where exogenous BR application was shown to boost the contents of chlorophyll a, b, and carotenoids under salt stress conditions [21]. Studies on Begonia pendula have revealed that 0.2 mg/L BR can mitigate the decline in photosynthesis, enhance chlorophyll concentration and Rubisco activity, and improve carbon dioxide assimilation and PSII electron transfer efficiency by regulating non-stomatal factors [22]. Maize research has confirmed that, in contrast to salt stress treatment, exogenous BR spraying reduces the malondialdehyde content. Regarding the antioxidant enzyme system, SOD and CAT activities increase and approach normal levels under combined treatment [23]. In this study, the ‘CC’ variety exhibited higher biomass accumulation, implying greater salt tolerance, whereas the ‘FD’ variety demonstrated lower biomass accumulation, suggesting lower salt tolerance. The robust salt tolerance of ‘CC’ is associated with its elevated levels of cellular proline, total free amino acids, and an efficient enzymatic antioxidant system. 

Exogenous spraying of BR can influence genes associated with the flavonoid synthesis pathway in tea plants. For example, genes related to the flavonoid synthesis pathway, namely *CsPAL*, *CsC4H*, *Cs4CL*, *CsCHS*, *CsF3H*, *CsF3*′*5*′*H*, *CsDFR*, *CsLAR*, and *CsANR*, were upregulated in tea plants upon BR treatment [13]. Simultaneously, exogenous BR spraying leads to an increase in theanine content and significantly upregulates theanine-synthesis-related genes, such as threonine synthase (TS) and glutamine synthetase (GS), in tea plants. Salt stress also markedly induces the expressions of genes in the theanine biosynthesis pathway, including *CsAlaDC*, *CsGOGAT1*, *CsGOGAT2*, and *CsTS1* [24]. These findings are consistent with those of this study. The underlying mechanism through which BR impacts osmotic adjustment substances is likely to span multiple hierarchical levels, as posited in references [25,26,27]. Li et al. [28] delved into an overexpression strain derived from the heterologous transformation of foxtail millet *SiRLK35* into rice, which was found to confer a certain degree of salt stress resistance. The *SiRLK35* gene product is capable of engaging in the salt stress response by modulating antioxidant enzyme activity and associated signaling pathways. Concurrently, BR is hypothesized to activate transcription factors via signal transduction cascades, thereby facilitating gene transcription. Moreover, it may exert an influence on the intracellular metabolic equilibrium, tilting it towards proline biosynthesis. The inhibitory effect of BR on proline degradation gene expression might impinge on post-transcriptional regulatory mechanisms, culminating in an augmented proline content that is instrumental in maintaining cellular osmotic pressure and functionality. Ethanol dehydrogenase stands as a pivotal enzyme within the constellation of plant aroma substance synthesis pathways, especially those intertwined with fatty acid metabolism, and it wields a substantial influence on the genesis of tea aroma compounds. Scholarly investigations have unearthed that the *CsADH1* protein is endowed with the catalytic capacity to convert (Z)-3-hexenal into (Z)-3-hexenol, thereby occupying a crucial niche in the biosynthesis pathway of tea tree hexacarbonyl volatile substances and, by extension, modulating tea aroma. The expression profile of the *CsADH1* gene exhibits pronounced variability across diverse tissues and under disparate environmental conditions in tea plants. Illustrative of this, in the autumnal season, the transcriptional activity of this gene in young stems is comparatively elevated; conversely, under low temperature exposure, its transcriptional level initially dips before rebounding [29]. These empirical observations resonate with the finding in the current study that *CsADH1* was upregulated under salt stress in tea plants, hinting at potential commonalities in regulatory mechanisms governing gene expression under stress. 

In summation, the pleiotropic effects of BR on chlorophyll content, photosynthetic efficiency, the antioxidative enzymatic system, osmotic regulators, and stress-responsive gene expression collectively constitute the linchpin factors underpinning enhanced salt tolerance in plants. The heterogeneous responses of diverse species and genes to BR administration bespeak the need for more in-depth exploration of its underlying mechanism. Consequently, it is of paramount significance to embark on comprehensive investigations into the molecular mechanisms and to finetune the application protocol of BR, with the overarching aim of bolstering plants’ resilience to salt stress and laying a solid foundation for future agricultural applications.

## 4. Materials and Methods

### 4.1. Plant Materials and Salt Stress Treatments

This experiment was carried out on the Chengdu Campus of Sichuan Agricultural University. The experimental materials selected were *Camellia sinensis* ‘Fuding Dabaicha’ (‘FD’) and ‘Chuancha 2’ (‘CC’), which are the principal cultivated varieties in Sichuan province. Healthy two-year-old tea plant cuttings were transplanted into plastic pots with a diameter of 28 cm and then cultivated in an experimental research garden at Sichuan Agricultural University (latitude 30°42′ N, longitude 103°51′ E, Chengdu city, China) under natural conditions. Geographically, this region falls within the humid monsoon climate zone of the mid-subtropical region, with an average temperature of approximately 15.9 °C, an annual rainfall of about 972 mm, an average of 12 sunshine hours per day, and an average relative humidity of 84%. Four tea seedlings were planted in each pot and cultivated in a soil environment for one year under regular cultivation management.

The tea seedlings used in the experiment were placed in a light culture room for one week and maintained at 25 °C with a light intensity of 200 μ mol m^−2^·s^−1^ for 14 h during the daytime and at 18 °C for 10 h during the night, along with a relative humidity of 70%. This experiment comprised three treatment groups: normal-growing tea seedlings (CK), salt-stressed tea seedlings (NA), and tea seedlings subjected to salt stress and treated with BR (NB). Among these treatment groups, the salt stress treatment was implemented by irrigating with 250 mL of NaCl solution at a concentration of 200 mM, whereas the control group was irrigated with an equal volume of distilled water. Three days later, a 0.2 mg/L BR solution was sprayed onto the corresponding seedlings, while the control group was sprayed with distilled water. In addition, three independent biological replicates were set up for each treatment group, and each replicate was sampled from 24 randomly selected tea seedlings. The tender leaves of tea plants were sampled at 0 days, 7 days, and 14 days after the application of BR and then immediately frozen in liquid nitrogen and stored at −75 °C until further analysis.

### 4.2. Determination of Physiological Indicators

Leaves were randomly sampled from the plants in each group for the measurement of their physiological characteristics. The determination of leaf chlorophyll concentration was carried out following the method described by Li et al. [30], with certain modifications, specifically using 95% ethanol for the extraction of photosynthetic pigments. The content of malondialdehyde (MDA) was measured in accordance with the method reported by Xiong et al. [31]. The contents of proline and soluble sugar were determined as described previously [30]. Meanwhile, the soluble protein content was extracted and analyzed following the protocol of Wang et al. [32]. The enzymatic antioxidant activities of superoxide dismutase (SOD), peroxidase (POD), and catalase (CAT) were determined using the Solepol kit (manufactured in Suzhou Grace Biotechnology Co., Ltd, Nanjing, China), which was based on the method of Li et al. [31] in accordance with the manufacturer’s instructions.

### 4.3. Total RNA Isolation and cDNA Synthesis

Total RNA was extracted from tea leaves by employing the EASY spin Plus Polysaccharide-Polyphenol Complex Plant RNA Rapid Extraction Kit (Adderall Biologicals, Beijing, China). The quality of total RNA was evaluated via 1.2% agarose gel electrophoresis and ultra-microUV spectrophotometry. First strand cDNA synthesis was synthesized using the PrimeScript™ FAST RT reagent Kit with gDNA Eraser (TaKaRa, Dalian, China) according to the manufacturer’s protocol.

### 4.4. Real-Time Quantitative PCR (qPCR) Detection of Genes Involved in Proline, Flavonoid, and Theanine Metabolism Pathway and Stress Response Markers

According to the results of Jin et al. [13] and Chen et al. [24], sixteen flavonoid-biosynthesis-related genes, including *CsPAL*, *CsC4Ha*, *CsC4Hb*, *Cs4CL*, *CsCHSa*, *CsCHSb*, *CsF3Ha*, *CsF3Hb*, *CsF3*′*5*′*Ha*, *CsF3*′*5*′*Hb*, *CsDFRa*, *CsDFRb*, *CsLAR*, *CsANRa*, *CsANRb*, and *CsANS*, four theanine-biosynthesis-related genes, theanine synthetase (*CsTSa* and *CsTSb*) genes, glutamine synthetase (*CsGSa* and *CsGSb*) genes, six proline-metabolism-pathway-related genes, including *CsProDH*-*A*, *CsProDH*-*B*, *CsP5CS*-*A*, *CsP5CS*-*B*, *CsOAT*-*A*, and *CsOAT*-*B*, and four stress-related genes, *CsRD29B*, *CsRD29A*, *CsRD20*, and *CsADH1*, were selected, and their specific primer sequences (Supplementary Material Table S1) in qRT-PCR were designed based on the Primer Premier 5.0 software. The TB tools-II [33] software is used to generate a heatmap for comparing the expression of genes related to flavonoid synthesis.

Quantitative PCR (qPCR) was carried out to assess the expression levels of genes implicated in the proline, flavonoid, and theanine metabolism pathway and stress response markers. The reaction system was designed according to the protocol of the 2X Realab Green PCR Fast Mixture Universal Kit (Lamboride, Beijing, China). The qPCR analyses were performed using SYBR Premix Ex Taq (Takara, Beijing, China) on the ABI PRISM 7900 HT Sequence Detection System with associated software from Applied Biosystems (Foster City, CA, USA) under the following cycling conditions: an initial preheating step at 95 °C for 30 s, followed by 40 cycles of denaturation at 95 °C for 10 s, annealing at 55 °C for 15 s, and extension at 72 °C for 10 s, with a final hold at 12 °C indefinitely. All experimental reactions were biologically replicated three times to ensure reproducibility. A melting curve analysis was conducted to detect any potential primer dimers. To standardize gene expression among different cDNA samples, the *GAPDH* gene from tea plants (GenBank Accession Number: KF027475) was employed as an internal control. The relative expression levels of relevant genes were calculated using the 2^−ΔΔCt^ method described by Schmitter et al. [34], and the significance of differences (*p* < 0.05) was analyzed by SPSS 26.0 software.

### 4.5. Statistical Analysis

Data were organized with Office Excel 2021 and TB tools-II, analyzed using SPSS 26.0, and multiple comparisons were carried out according to the Waller–Duncan method. Differences between treatments were compared based on the classification of treatment time, where different letters denote a significance level of *p* < 0.05. Graphs were generated using Graph Pad 8.0.2.

## 5. Conclusions

This study was designed to comprehensively investigate the impacts of exogenous BR spraying on the levels of malondialdehyde, soluble sugar, and soluble protein and the activities of antioxidant enzymes in tea plants under salt stress conditions. Additionally, it delved into the expression alterations of genes associated with the synthesis pathways of proline and secondary metabolites, namely flavonoids and theanine. It was hypothesized that various physiological indicators of tea plants would exhibit an upward trend under salt stress as a defense mechanism against salt-induced damage. Under salt stress, the genes associated with the synthesis pathways of proline and secondary metabolites (flavonoids and theanine) were upregulated; concomitantly, the genes responsible for proline degradation were downregulated. These findings suggest that when tea plants are exposed to salt stress, the expression of genes related to the synthesis of secondary metabolites is adaptively regulated to counteract the adverse effects of salt stress. Moreover, exogenous BR spraying evidently enhanced the salt tolerance of tea plants, as manifested by the modulated gene expression patterns.

## Data Availability

All the data presented in this study are included in this manuscript and Supplemental Materials.

## Supplementary Materials

The following supporting information can be downloaded at: https://www.mdpi.com/article/10.3390/ijms252413445/s1.
