# Supplementary material for: Effects of Brassinosteroid on the Physiological Changes on Two Varieties of Tea Plants Under Salt Stress
Source: Int J Mol Sci. 2024 Dec 15;25(24):13445. doi: 10.3390/ijms252413445 (PMC11677880; doi:10.3390/ijms252413445)
Supplement: Supplementary file 1 [file ijms-25-13445-s001.zip › ijms-3333669-supplementary.pdf]

**Table S1 Primer sequences**

| Name of gene | Forward primer/F | Reverse primer/R |
|---|---|---|
| *CsRD29B* | ACTGGAATCGCAAACAAG | GACAGAAAACCCCAACAA |
| *CsRD29A* | AGAACCAAATCAAACAGACC | AACGAGGAAGGTTGAGAA |
| *CsRD20* | GCTTTGAGTTACCCTACA | GTCATAAGTTGCGGAGTC |
| *CsADH1* | AGAGTGTCTGGTGCTTCG | TTGTGGTCTTTGGGGTTC |
| *CsPDH-A* | CAAAACCCAAATCCAACC | GTCCACCATCGGCTCTAT |
| *CsPDH-B* | GTGGAATGCTGGTTTACG | AGATCGCTTACTCGCTGT |
| *CsP5CS-A* | GGCTCGCTATGAACAATC | CCTTTCAAGAGTAACCCATT |
| *CsP5CS-B* | TTGCTGAGCGATGTAGAC | TAAGAGGAAGCCACGAAT |
| *CsOAT-A* | GGTGCCGAAGGTGTAGA | CAAAACCACGAGTAGCC |
| *CsOAT-B* | TAGAACAGGCAGAAAGG | ATAGCCAATGTACGACC |
| *CsPAL* | CCGTTCAAGCAAGCAGT | ACATTGTAGCCCTCGTAGA |
| *CsC4Ha* | CGATAGAATGGGGCATAGCA | TGGAGGTAGGGGAGTTTGTAGG |
| *CsC4Hb* | GCTCGGCAGCTATGACATCC | CTCCTCCTACCAACACCGAATG |
| *Cs4CL* | AGCGGCATAACTATTACCACAGC | TCCCGACGAATAAGGTAGCG |
| *CsCHSa* | TGAAGGACCTGCCACGGTTATG | GCCTTATGCTCGCTGTTTGT |
| *CsCHSb* | GGCAACCCCACCAAACT | CCACCTTATGCTCGCTATTA |
| *CsF3Ha* | ACAACAACGCTTACGGCTCTC | AAACCCCCCAACCTTCACA |
| *CsF3Hb* | TCAAACGCCACACAGACCC | CAACGAAAGCCCCCTCAA |
| *CsF3'5'Ha* | ATCCTCTCTAAACCCCTCC | ATCCCACAAGTGCCCAT |
| *CsF3'5'Hb* | CAAAAAAGTCTTAGCCGTCGC | CTCTCAATCCCTTGTAAGTCCATC |
| *CsDFRa* | ATTGGCAGAGAAAGCAGCAT | GTGATTAGGCTTGGTGGGAA |
| *CsDFRb* | AATGGTTATGGTGGTGGCG | CGAAATTCATGGGAGTGGC |
| *CsLAR* | AAAAGAGGAGGGTGCGG | GGAACTCATCCAAAGGGGG |
| *CsANRa* | GAGTACTTCAAGGCTAAGGGG | CAAGCAAACCAAGCAAAACC |
| *CsANRb* | CTGGCAATCCAAGGAGTGC | GCCCCGTTCCATCAAGC |
| *CsANS* | GTTTTCTGCGAACCACCCA | TTTTCCTCCAAGCACCTGAGT |
| *CsTSa* | AGACCGCCGACATCAACAC | ATGGCTTCCACAGCAGAGT |
| *CsTSb* | CCTAAACCTATTGAGGGTGACTG | TCCTGTAAGCCGACGCTCATT |
| *CsGSa* | ACAAGTTAAGTGGCCGCTTG | TCGCGTCCATAAGCTTTGTC |
| *CsGSb* | AAGGCCATCGAAAAGCTTGG | TCAGCTGTTTCGTGCTTTCC |
| *CsGAPDH* | TTGGCATCGTTGAGGGTCT | CAGTGGGAACACGGAAAGC |
